# Screening of Native Plants Growing on a Pb/Zn Mining Area in Eastern Morocco: Perspectives for Phytoremediation

**DOI:** 10.3390/plants9111458

**Published:** 2020-10-29

**Authors:** Said El Hasnaoui, Mouna Fahr, Catherine Keller, Clément Levard, Bernard Angeletti, Perrine Chaurand, Zine El Abidine Triqui, Abdelkarim Guedira, Laila Rhazi, Fabrice Colin, Abdelaziz Smouni

**Affiliations:** 1 Laboratoire de Biotechnologie et Physiologie Végétales, Centre de Biotechnologie Végétale et Microbienne Biodiversité et Environnement, Faculté des Sciences, Université Mohammed V de Rabat, 10000 Rabat, Morocco; said.elhasnaoui@um5s.net.ma (S.E.H.); z.triqui@um5s.net.ma (Z.E.A.T.); a.guedira@um5s.net.ma (A.G.); 2 Laboratoire Mixte International Activité Minière Responsable “LMI-AMIR”, IRD/UM5/INAU, 10000 Rabat, Morocco; keller@cerege.fr (C.K.); levard@cerege.fr (C.L.); angeletti@cerege.fr (B.A.); chaurand@cerege.fr (P.C.); fabrice.colin@ird.fr (F.C.); 3 Aix Marseille Univ., CNRS, IRD, INRAE, Collège de France, CEREGE, 13100 Aix-en-Provence, France; 4 Centre de Biotechnologie Végétale et Microbienne Biodiversité et Environnement, Faculté des Sciences, Université Mohammed V de Rabat, 10000 Rabat, Morocco; laila.rhazi@um5.ac.ma

**Keywords:** phytoextraction, phytostabilization, native plants, heavy metal, hyperaccumulation, tolerance, Pb/Zn mining area

## Abstract

Screening of native plant species from mining sites can lead to identify suitable plants for phytoremediation approaches. In this study, we assayed heavy metals tolerance and accumulation in native and dominant plants growing on abandoned Pb/Zn mining site in eastern Morocco. Soil samples and native plants were collected and analyzed for As, Cd, Cu, Ni, Sb, Pb, and Zn concentrations. Bioconcentration factor (BCF), translocation factor (TF), and biological accumulation coefficient (BAC) were determined for each element. Our results showed that soils present low organic matter content combined with high levels of heavy metals especially Pb and Zn due to past extraction activities. Native and dominant plants sampled in these areas were classified into 14 species and eight families. Principal components analysis separated *Artemisia herba-alba* with high concentrations of As, Cd, Cu, Ni, and Pb in shoots from other species. Four plant species, namely, *Reseda alba*, *Cistus libanotis*, *Stipa tenacissima*, and *Artemisia herba-alba* showed strong capacity to tolerate and hyperaccumulate heavy metals, especially Pb, in their tissues. According to BCF, TF, and BAC, these plant species could be used as effective plants for Pb phytoextraction. *Stipa tenacissima* and *Artemisia herba-alba* are better suited for phytostabilization of Cd/Cu and Cu/Zn, respectively. Our study shows that several spontaneous and native plants growing on Pb/Zn contaminated sites have a good potential for developing heavy metals phytoremediation strategies.

## 1. Introduction

Soil contamination by heavy metals has become one of the major environmental problems around the world [1]. Anthropogenic activities such as mining, smelting, and industrial processing are the most important sources of heavy metals entering the environment [2,3,4]. Heavy metals persist a long time in the soils as these elements are not biodegradable [2,5]. This contamination affects the taxonomic, functional diversity, and microbial properties of soils [6]. Moreover, due to their high concentrations in soils, metals can be transferred and accumulated not only in the plants [7], but also in the humans being, causing many serious health disorders [8]. There are several technologies to remediate heavy metals contaminated soils (e.g., excavation of contaminated material and chemical/physical treatment). However, many of these technologies are costly or do not achieve a long-term nor aesthetic solution [9,10].

Phytoremediation is a so-called “green” and “cost-effective" technique [4] whereby plant species are used to detoxify contaminated soils through immobilization or hyper/accumulation processes [11,12]. The remediation of toxic metals by plant can be divided into two most important strategies: (1) phytostabilization: plants are used to stabilize contaminated soil from the potential migration of pollutants by immobilizing contaminants in root system through absorption into the roots or precipitation in the rhizosphere [13]; and (2) phytoextraction: plants are used to remove metal pollutants from soils via root absorption and their concentration in the shoots and leaves [4,14]. Phytoextraction uses both accumulator and “hyperaccumulator” plants. These latter can accumulate unusual amounts of metals (>1% and up to 10%) in their shoots [14,15]. However, plants adaptation to environmental conditions of target regions is among factors limiting the success of the phytoremediation strategy [11]. Spontaneous and native plant species could overcome these constraints, constituting a key element in the efficiency of phytoremediation strategies. The native plants are often efficient in terms of growth, reproduction, and survival under environmental stress [16]. In this way, there is a continuing interest in searching for native plants that are tolerant to heavy metals and can promote phytoremediation [12].

The screening of spontaneous and native plant species from mining sites all over the world becomes a subject of interest. In Morocco, there are many natural metalliferous sites and metal-contaminated soils, and their metallophyte flora still poorly explored. Oued el Heimer and Touissite sites in eastern Morocco are among the most demonstrative examples of abandoned mining areas. After overexploitation during more than 70 years [17], they are a source of contamination by heavy metals because of poorly controlled waste disposal. These mining areas show an adapted native flora that has the potential to be used for different phytoremediation strategies to rehabilitate these contaminated areas. The objectives of this study are (i) screening of native plants growing on a contaminated area in the vicinity of a Pb/Zn mining area in eastern Morocco; and (ii) identification of tolerant and/or hyperaccumulator plants to suggest suitable plants for use in phytoremediation.

## 2. Materials and Methods

### 2.1. Sample Collection

**Figure 1:**
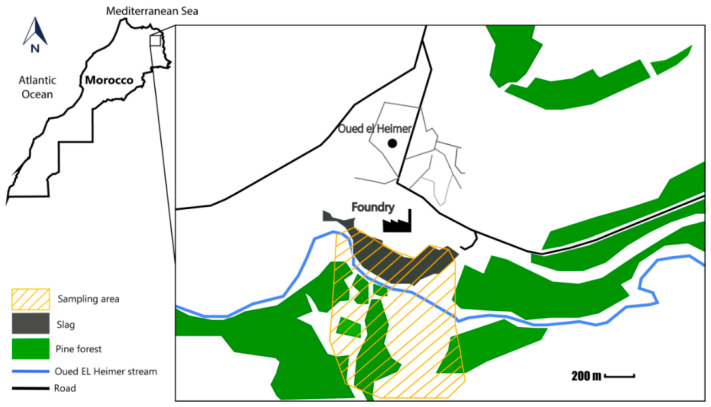
Plant and soil sampling area at Oued el Heimer site.

**Figure 2:**
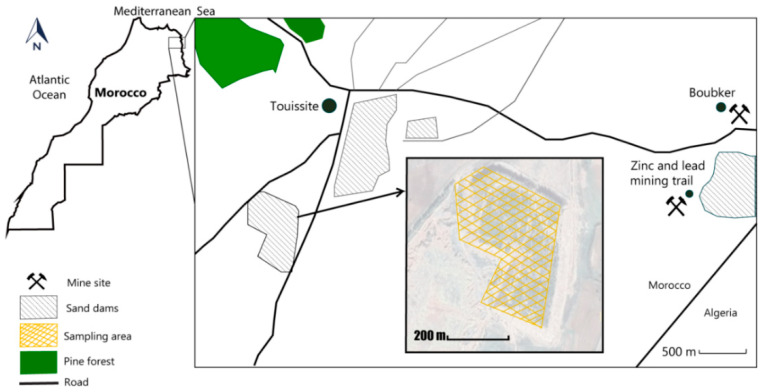
Plant and soil sampling area at Touissite site.

Soil and plant sampling were collected in the “Oued el Heimer (34°26′50.5″ N 1°54′07.3″ W) and Touissite (34°28′07.7″ N 1°46′20.9″ W)” areas during May 2018. Eighty-four samples of dominant plants were collected, (about 6 species from Oued el Heimer and 8 from Touissite with 6 replicas per species). To obtain the accurate plant species information, plants were identified according to flora of Morocco [18,19,20]. Soil samples, from the plants rooting zone (0–20 cm depth), were also collected. Seventy-eight samples of soils were collected (about 6 samples from Oued el Heimer and 7 from Touissite with 6 replicas per sample) (Figure 1 and Figure 2).

### 2.2. Soil and Plant Analysis

Soil samples were air-dried and sieved to <2 mm powder before use. The percentage of organic matter (OM) content were determined according to the method described by Combs et al. [21]. Soil pH was determined in 5:1 water/soil. The mixture was stirred for 1 h with a magnetic stirrer and then decanted for 30 min. The pH was determined on the supernatant directly using a pH meter “Bante210 Benchtop pH/mV Meter, Bante instruments CO., LTD. Shanghai, China” [17]. Electrical conductivity (EC) (1:5 *w*/*v* soil water suspensions) was carried out using a multi-parameter measuring instrument “MultiLine^®^ Multi 3510 IDS, WTW a xylem brand”. For heavy metal content analysis, soil samples were dried at 72 °C to constant weight for two days and digested with a mixture following this procedure: 100–300 mg of soil material is placed in Polytetrafluoroethylene (PTFE) tubes. The soil samples are first moistened with 0.2 mL of water and then in order, 2 mL of concentrated HCl (37%, A Fisher Scientific International Company Pittsburgh, Pennsylvania, USA) and 1 mL of concentrated HNO_3_ (69%, Loba Chemie PVT. LTD Colaba, Mumbai, INDIA) are added. The mixture is incubated for 16 h at room temperature. The mixture is then brought to the boil for 2 h at 150 °C by placing a glass bead above each tube for reflux. After dissolving the soil sample, the mixture is transferred quantitatively to a 15 mL tube, adjusting with distilled water [22]. Plant samples were separated into shoots and roots, washed thoroughly with running tap water to remove adhering substrate materials, rinsed twice for 15 s in cold 0.2 mM CaSO_4_, and rinsed with cold distilled water. The plant samples were dried at 72 °C for 48 h and grounded to powder. Then, plant samples were digested according to the acid hydrolysis protocol described by Temminghoff and Houba [22]. The concentrations of As, Cd, Cu, Ni, Sb, Pb, and Zn in digested soil or plant samples were then analyzed in ICP-AES (inductively coupled plasma–atomic emission spectrometry; Ultima2 JY, Horiba Company, Edison, NJ, USA) according to Margui et al. [23] with the blank values being deducted from the measurement.

### 2.3. Soil Pollution Index (PI)

The pollution index of soils is calculated by dividing the metal concentrations in soil (mg kg^−1^) by the background metal concentration according to Chon et al. [24] and Wu et al. [25]. This background concentration corresponds to standard levels in uncontaminated soils [26]. Thus, the pollution index is calculated using the following equation:

PI = ([As]_soil_/6 + [Cd]_soil_/3 + [Cu]_soil_/100 + [Ni]_soil_/100 + [Pb]_soil_/100 + [Zn]_soil_/300 + [Sb]_soil_/8.6)/7 [metal]_soil_: concentration in mg kg^−1^ (1)

where a PI greater than 1 corresponds to a polluted soil.

### 2.4. Soil Enrichment Factor (EF)

The enrichment factor (EF) is used to assess the impact of human activities on the natural environment. It is calculated by the following equation [27]:

EF = (C_i_/C_ref_) _sample_/(C_i_/C_ref_) _background_ (2)

where (C_i_/C_ref_) sample is the ratio of the content of the interested element and reference element in the soil sample, and (C_i_/C_ref_) background is the ratio of the content of the interested element and reference element in natural background (Average Crust) [26] (Table S1). In this study, Scandium (Sc) was selected as a reference element [28,29].

According to Liu et al. [30] five contamination categories are generally recognized based on the enrichment factor: EF < 1, no enrichment; 1 ≤ EF < 10, minimal enrichment; 10 ≤ EF < 100, moderate enrichment; 100 ≤ EF < 1000, significant enrichment; and EF > 1000, extremely high enrichment.

### 2.5. Bioconcentration Factor, Translocation Factor, and Biological Accumulation Coefficient

To evaluate the metal enrichment characteristics of different plant species, three factors were calculated:

The biological concentration factor (BCF) is found by dividing the plant root metal concentration by the metal concentration in rhizospheric soils (Table S1) [31]. This factor shows the accumulation ability for root from the soil. It is calculated using the following formula:

BCF = [Metal] root/[Metal] soil(3)

Translocation factor (TF) is the metal ratio transfer capacity from root to shoots (Table S1) [31]. It is calculated using the following formula:

TF = [Metal] shoot/[Metal] root(4)

Biological accumulation coefficient (BAC) is defined as the concentration of metals in plant shoots divided by metal concentration in rhizospheric soils (Table S1) [32]. It is calculated using the following formula:

BAC = [Metal] shoot/[Metal] soil(5)

### 2.6. Data Analysis

Differences in metal concentrations in roots and shoots among species were studied using one-way ANOVA. This analysis was made separately for each metal in the shoots and the roots. To identify the significant pair-wise differences between species for each metal concentration, post hoc Tukey HSD tests were used.

To study the relations between metals concentrations in shoots and roots for the studied plant species, principal component analysis (PCA) was performed. The data matrix contains the average values (6 replicates) of each metal in the shoots and in the roots of each species. The similarity between species in the ability of metal accumulation in shoots and roots was studied using ascending hierarchical classification (AHC).

Multivariate and univariate analyses were carried out using STATISTICA 10 (StatSoft Inc., Tulsa, OK, USA).

## 3. Results and Discussions

### 3.1. Site Description

The studied area “Oued el Heimer—Touissite” is located in the North-West of Morocco, at the mountainous band (Horst chain), which stretches over a length of 100 km and a width of 20–30 km. This chain is North limited by the corridor Oujda-Taourirt, on the West by the plain of Tafrata, in the South by the vast domain of high plateaus, and in the East by the Morocco–Algerian border [17]. Semi-arid Mediterranean climate conditions with a dry and hot summer of variable duration and wet winter prevail in this area. Annual rainfall rates range from 90 to 100 mm/year [33].

Oued el Heimer foundry is close to the village of Oued el Heimer, which is located approximately 33 km southeast of Oujda town. This smelter, closed in 2013, specialized in the treatment, fusion, and refining of Pb and silver (Ag). Oued el Heimer area is subjected to intense pollution by a massive accumulation of hazardous wastes in the neighborhood of the smelter. These wastes form slag particle mounds at the banks of the Oued el Heimer creek. In the period of floods, waters carry important quantities of wastes. This natural leaching constitutes a significant source of contamination of grounds and water sources (Figure 1) [17].

The Touissite mining site, located thirty kilometers south the Oujda city, was closed in 2002, following 75 years’ period of exploitation. Source of pollution in these areas originates mainly from mining waste which forms sandy dams on large surfaces. These sands are easily transported by winds and rainfall. Some of these dams were covered by residual materials directly stemming from the ore extracting process (Figure 2) [17].

**Figure 3:**
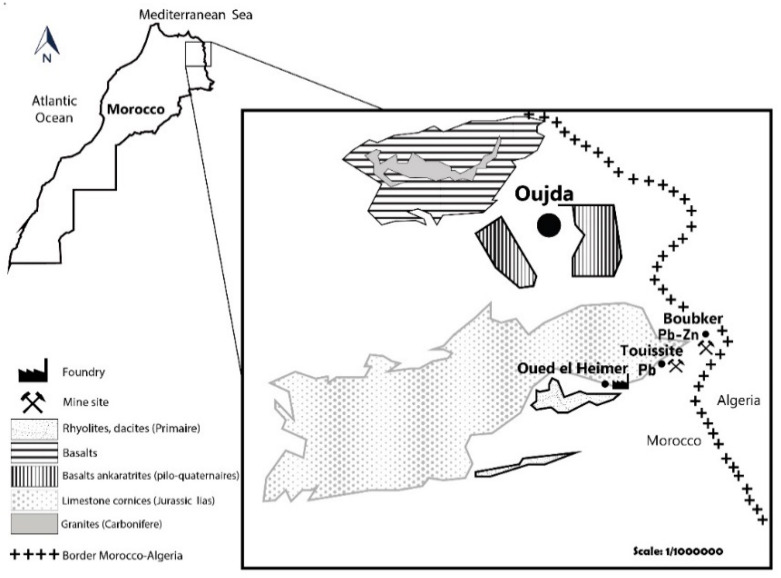
Geology of the Touissite area (Ministry of energy and mines geology department, 1985).

The Pb–Zn ores of the Touissite area substitute recrystallized Liassic dolomites at the base of a block-faulted Jurassic section, which rests on Paleozoic basement rocks. Galena, sphalerite with pyrite and marcasite dominate the mineral assemblages with lesser chalcopyrite, bornite, and tetrahedrite. Pb–Sb sulphosalts (bournonite and stephanite) and Ag-bearing species (argentite and native silver) are rather scarce [34,35,36] (Figure 3).

### 3.2. Soil Properties and Metals Concentrations

The soils were slightly acidic to neutral with a pH ranging from 6.45 to 7.51 at the Oued el Heimer site and from 6.91 to 7.76 at Touissite (Table 1). These results confirm the data reported by Smouni et al. [17] related to soils of the same area of Oued el Heimer. Midhat et al. [8] report pH values of 7.4 in soils of Sidi Bou-Othmane mining site located in Marrakech (Morocco). Navarro et al. [37], however, found pH values ranging from 3.1 to 8.4 in the Pb–Zn mined areas of Cabezo Rajao in Spain. Many authors have shown that soil pH plays a crucial role in the solubility and bioavailability of metals [38]. Bliefert and Perraud [39] reported that solubility of Pb decreases with increasing pH. Compared to pH, EC values showed greater variability ranging from 0.40 to 2.28 and from 0.23 to 0.38 mS/cm respectively at Oued el Heimer and Touissite. These values indicate low salinities of both studied site soils [40]. Soil samples from Oued el Heimer presented a low organic matter (OM) content that varies respectively between 0.41% and 2.80%. The same trend was observed for soil samples from the Touissite site with 0.28% to 2.07% for organic matter (Table 1). Low OM contents reflect low biological activities and plant developments. These may be due to specific environmental conditions combined to high amount of heavy metals in soils. Indeed, some authors suggest that high levels of Zn in soils, negatively influence the activity of micro-organisms and earthworms, thus inhibiting the breakdown of organic matter [41].

Heavy metals concentrations in soils sampled from Oued el Heimer and Touissite sites are presented in Table 1. The soils from Touissite site have As concentrations varying between 43 and 82.90 mg kg^−1^ dry weight (DW), between 15 and 36 mg kg^−1^ DW for Cd, 328 and 1405 mg kg^−1^ DW for Cu, 9 and 16 mg kg^−1^ DW of Ni, 6445 and 18,324 mg kg^−1^ DW of Pb, 96.71 and 242.80 mg kg^−1^ DW of Sb, and between 2096 and 5385 mg kg^−1^ for Zn. The area of Oued el Heimer, where the smelter is located, has a content of As ranging between 18.7 and 466 mg kg^−1^ DW, Cd from 32 to 280 mg kg^−1^ DW, Cu from 35 to 592 mg kg^−1^ DW, Ni between 12 and 44.4 mg kg^−1^ DW, Pb between 611 and 12,461 mg kg^−1^ DW, Sb from 33.90 to 247.10 mg kg^−1^ DW, and Zn between 318 and 43,540 mg kg^−1^ DW. This area is characterized by high Zn and Pb levels. Therefore, soils from these areas can be considered as highly contaminated [26]. Pollution index (PI) recorded at both sites ranged from 3.62 to 67.90 at Oued el Heimer and from 14.00 to 38.51 at Touissite (Table 1). This expresses the extremely deleterious effect of these mining activities and abandoned wastes on the environment.

Enrichment factor (EF) defines the concentrations of a given element at a specific location as compared to the average natural occurrences [42]. In Oued el Heimer site, the highest EF values for As, Cd, Cu, Ni, Pb, Zn, and Sb were 364.8, 3656.4, 98.8, 8.2, 2995, 2749.6, and 2393.3, respectively (Table 1). At Touissite mine, the highest enrichment factor (EF) for As, Cd, Cu, Ni, Pb, Zn, and Sb were respectively 52.5, 410.4, 166.5, 2.3, 2758.3, 1092.2, and 1877.8, (Table 1). According to EF categories [30], all metals in both areas were affected by human activities. Among them, the EF values of Ni at both sites are less than 10 (1 ≤ EF < 10), which was moderately enriched and affected both by a natural source and human activities [30]. The enrichment factor values for Cu (Oued el Heimer site) and As (Touissite site) belonging to category (10 ≤ EF < 100), indicate moderate enrichment. The EF values for As (Oued el Heimer site), Cd, and Cu (Touissite site) belonging to category (100 ≤ EF <1000), indicate that As, Cd, and Cu were significantly enriched.

The EF values for Cd at Oued el Heimer site and Pb, Zn, and Sb at both sites attached to category (EF > 1000), indicate extremely high enrichment, and these values revealed that heavy metals contents are affected mainly by anthropogenic activities (Human factors) [30].

Many reports recorded different degrees of soil metal concentrations in the vicinity of mining sites and smelters. For example, Navarro et al. reported 19 and 53.1 mg kg^−1^ DW of Pb and Zn, respectively, in Cabezo Rajao abandoned mine (Pb–Zn) in SE Spain [37]. El Hachimi et al. [43] reported the same trend of heavy metal contents in some Pb and Zn mining site in High Moulouya from Morocco, 5547 mg Pb kg^−1^ and 7500 mg Zn kg^−1^ at Zaida mine, 10,520 and 9075 mg kg^−1^ of Pb and Zn, respectively, at Mibladen mine, and 2101 mg kg^−1^ of Pb and 3125 mg kg^−1^ of Zn at Aouli. Furthermore, Yassir et al. [40] reported 321.7 and 723.4 mg kg^−1^ of Pb and Zn, respectively, at Sidi Bou Othmane abandoned mine (Marrakech, Morocco). The concentrations of Pb and Zn in the Ahangaran mine at Malayer City, South of Hamedan in Iran, are respectively about 8955.4 and 12,963.1 mg kg^−1^ DW [44]. The Pb and Zn concentrations found in Oued el Heimer and Touissite were much lower than those reported in two mining districts (Maline and Les Avinières) located in Southern France (more than 84,130 mg Pb kg^−1^ and 91,454 mg Zn kg^−1^) by Escarré et al. [45]. Other studies reported approximately the same range of As, Cd, and Cu recorded in soils from our study [37,40]. For Antimony (Sb), many reports recorded 100.6–5045 mg kg^−1^ DW [46], 527–11,798 mg kg^−1^ DW [47], and between 74.2 and 16,389 mg kg^−1^ of Sb [48] in different mining sites in China. Moreover, about 2900 mg kg^−1^ of Sb were measured at Sb smelting sites in Japan [49]. According to previous studies, the majority of abandoned mines present high levels of soil metals contamination due to anthropogenic activities. We report similar results. In this context, identification of native plants and characterization of their heavy metals tolerance and accumulation potential may offer efficient solutions for contaminated area restoration.

### 3.3. Screening of Native Plant Species in Oued el Heimer and Touissite Areas

A total of 14 plant species were identified and classified to 8 families according to the nomenclature of “Flore Pratique du Maroc” [18,19,20] (Table 2) with a dominance of the Brassicaceae, Fabaceae, and Asteraceae families. At Oued el Heimer site, six samples were collected downstream of the el Heimer Oued in the area located 500 m far from the smelter and surrounded by pin forest and identified as *Cistus libanotis, Artemisia herba-alba,* and *Capsella bursa-pastoris* belonging to Cistaceae, Asteraceae, and Brassicaceae families, respectively. Other samples taken directly from the slag were identified as *Hirschfeldia incana, Stipa tenacissima* and *Agathophora alopecuroides* belonging respectively to Brassicaceae, Poaceae, and Amaranthaceae families. In the Touissite site covered by mining waste, eight samples were identified belonging to eight species: *Reseda alba, Convolvulus althaeoides, Hedysarum spinosissimum, Phragmites communis, Lotus corniculatus,* *Capsella bursa-pastoris*, *Scolymus hispanicus* L., and *Rapistrum rigosum*. The sampled plant species were divided into two life-forms, perennial and annual according to the flora of Morocco [18,19,20]. All plant species belong to the most important groups of worldwide spontaneously distributed in the Mediterranean region [50,51,52,53,54,55,56,57]. Many authors reported their occurrence in both uncontaminated and contaminated soils, including mining sites ecosystems. For example, several species of Brassicaceae, Cistaceae, Fabaceae, and Resedaceae families grow naturally in Pb–Zn and Sb contaminated soils in Spain [58,59]. Furthermore, Asteraceae, Poaceae, and Amaranthaceae are found in the vicinity of different mining sites in southern central Morocco [8]. The majority of these families could also be found at other mining sites located outside of the Mediterranean region such as in 18 metalliferous areas of the Austrian Alps [60], in the lead–zinc mine in Iran [44,61,62], in the gold mining site, in Colombia [63], in Zn rich mining soils in China [41], in Pb–Zn mining areas in Vietnam [64], in a metal-contaminated site in Florida [16] and in As-contaminated soils in the Czech Republic [65].

### 3.4. Heavy Metals Concentration in Plants

Metal roots and shoots contents are given in Table 3 that also provide phytotoxic levels and hyperaccumulation threshold issued from Kabata-Pendias and pendias [66]; Kramer [67] and Bioconcentration factors (BCF), translocation factors (TF), and biological accumulation coefficient (BAC) in Table 4.

#### 3.4.1. Arsenic (As)

Arsenic (As) total concentrations in the roots and shoots was significantly different between species (Table 3 and Table 4). As contents in the roots of *Stipa tenacissima* and *Artemisia herba-alba* were significantly (*p* < 0.05) higher as compared to those in the other plant species. While *Rapistrum rigosum* accumulate the low concentration of this metal (1.3 mg kg^−1^ DW). In shoots, As amount in shoots of *Artemisia herba-alba* followed by *Stipa tenacissima* and *Cistus libanotis* were significantly higher compared to other species. *Artemisia herba-alba* present significantly the highest value up to 50.7 mg kg^−1^ DW while the lowest accumulation of As was found in *Rapistrum rigosum* (0.3 mg kg^−1^ DW). Two plant species from the Oued el Heimer site reach the maximum values of As in their roots and shoots, including *Stipa tenacissima* and *Artemisia herba-alba*. However, all plant species collected around the investigated sites accumulated As concentrations less than 80 mg kg^−1^ in their roots and shoots, which is a phytotoxic As concentration in plants [67]. Arsenic hyperaccumulators are defined as plants that accumulate more than 1000 mg kg^−1^ DW in their shoots [68]. In this study, none of the 14 plant species showed Arsenic (As) concentrations in their tissues (Table 3) that exceed these thresholds; thus, none of them is As hyperaccumulators.

Several authors used BCF, TF, and BAC to evaluate the metal accumulation efficiency in plants and to estimate the plants potential for phytostabilization and/or phytoextraction [69,70]. BCF, TF, and BAC with values higher than 1 are used as criteria to select the plants used for phytoextraction, while BCF > 1 and TF < 1 had been used to evaluate the potential of plants for phytostabilization [71]. None of the collected plant species has BCF > 1 (Table 5). However, nine of them have a TF slightly >1 such as *C. libanotis* (2.87), *R. alba* (2.37), *C. althaeoides* (2.12), *C. bursa-pastoris* (1.53) from Touissite and *S. hispanicus* L. (1.32), *P. communis* (1.31), *C. bursa-pastoris* from Oued el Heimer (1.13), *H. incana* (1.10), and 1.04 in *A. herba-alba*. All plant species have a BCF and BAC < 1 (Table 5) indicating that none of the fourteen plant species has the potential to be used in As phytostabilization (BCF > 1 and TF < 1) or for As phytoextraction (BCF, TF, and BAC > 1).

#### 3.4.2. Cadmium (Cd)

The Cd content in shoots and roots were significantly different between species (Table 3 and Table 4). Cd content in roots of *Stipa tenacissima* up to 241.2 mg kg^−1^ DW was significantly much higher than those observed in other plants (Table 3 and Table 4). While the lowest accumulation of Cd was recorded in *R. rigosum* (1.7 mg kg^−1^ DW) in roots. Midhat et al. [8] indicate that the plants from *Stipa* genus, such as *Stipa capensis Thunb* accumulates 7.82 mg kg^−1^ DW in their root, which means that *Stipa tenacissima* is characterized by a strong capacity to take up Cd from the soil compared to other species from the same genus. In shoots, the highest concentrations of Cd are found significantly (Table 4) in *Artemisia herba-alba* followed by *H. incana,* *C. bursa-pastoris, S. tenacissima, A. alopecuroides,* and *C. libanotis* harvested from Oued el Heimer site compared to those in the other plant species and reach respectively 56.1, 32.9, 30.0, 28.9, 18.0, and 16.2 mg kg^−1^ DW (Table 3). Many reports recorded various amounts of Cd in shoot parts such as 0.62 mg Kg^−1^ DW in *Artemisia vulgaris* growing near a mining site in Northern Vietnam [72], 3.28 and 4 mg kg^−1^ DW in *Stipa capensis Thunb* and *Stipa barbata Desf,* respectively, growing around lead–zinc mine [8,73]. Plants generally exhibit measurable Cd amounts, especially in roots, but also in shoots resulting from uptake and translocation [8]. According to Kramer [67], *R. alba, H. spinosissimum,* *C. bursa-pastoris* (Touissite), *S. hispanicus* L., *C. libanotis*, *A. alopecuroides*, *H. incana*, *S. tenacissima*, *A. herba-alba*, and *C. bursa-pastoris* (Oued el Heimer) accumulate more than the Cd phytotoxic concentrations (0.1 to 3 mg kg^−1^) in plant and are considered Cd tolerant species. A shoots concentration higher than 100 mg kg^−1^ is used as a criterion for Cd hyperaccumulation [68]. On this basis, none of the 14 plant species can be considered as Cd hyperaccumulator.

The BCF for Cd varied from 0.07 in *P. communis* to 2.72 in *S. tenacissima* (Table 5). Five plant species have a TF > 1, namely *A. alopecuroides* (3.67), *R. alba* (1.91), *H. incana* (1.27), *A. herba-alba* (1.11), and *S. hispanicus* L. (1.03) (Table 5). The values of the translocation factor are higher than the bioconcentration factor. This indicates that plants translocate Cd from roots to shoots [16]. The value of biological accumulation coefficient (BAC) higher than one is recorded only in *A. herba-alba* (1.10) (Table 5). Based BCF, TF, and BAC values, none of the plant species has the potential to be used in Cd phytoextraction [14]. *S. tenacissima* has BCF = 2.72 and TF = 0.11, also showed a high accumulation of Cd in its roots, which suggests that it is a suitable plant for Cd phytostabilization. This native plant species with both the capacity to accumulate high amounts of Cd in its roots and high biomass production could be used to minimize the migration of Cd in soil. This process reduces metal mobility and leaching towards groundwater’s [74].

#### 3.4.3. Copper (Cu)

Among the collected plants, Cu content in plants range from 10 mg kg^−1^ DW (*R. rigosum* shoot) to 237.8 mg kg^−1^ DW (*S. tenacissima* root). Cu contents in roots of *S. tenacissima* and *A. herba-alba* were significantly higher as compared to those in the other species, whereas the concentrations in shoots were found to be significantly (*p* < 0.05) higher in *A. herba-alba*, *P. communis,* and *C bursa-pastoris* (from Touissit area) compared to other plant species (Table 3 and Table 4). Other studies reported 23.42 mg kg^−1^ DW in *Scolymus hispanicus* L. and 0.07 mg kg^−1^ DW in *Convolvulus althaeoides* [8]. This variation of accumulation of Cu depends in part upon the soils physicochemical proprieties [75]. Nine plant species, namely *R. alba, C. althaeoides, H. spinosissimum, P. communis, L. corniculatus,* *C. bursa-pastoris* (Touissite), *S. hispanicus* L., *S. tenacissima*, and *A. herba-alba,* accumulate Cu above phytotoxic concentrations (20–30 mg kg^−1^) in the plant tissues, which suggested that they are tolerant to Cu [67]. Copper hyperaccumulator plants are defined as plants that accumulate more than 1000 mg Cu/kg DW in aboveground tissues [76]. On this basis, none of the collected plant species can be considered as Cu hyperaccumulators.

The lowest BCF value was observed in *P. communis* (0.04) and the maximum BCF values were measured in the tissues of *A. herba-alba* (3.63) and *S. tenacissima* (1.55) (Table 5). The TF ranges from 0.12 in *S. tenacissima* to 1.08 in *C. libanotis*. For the biological accumulation coefficient, only *A. herba-alba* presented a BAC equal to 1.29. According to these values, none of the 14 plant species has the potential for Cu phytoextraction from the soil. However, the BCF was >1 and TF < 1 in both *A. herba-alba* and *S. tenacissima.* These plant species showed a high ability to tolerate and accumulate Cu in roots and are then suggested suitable for Cu phytostabilization.

#### 3.4.4. Nickel (Ni)

In the present study, all plant species showed a low Ni content in their roots ranged between 1.3 mg kg^−1^ DW in *R. rigosum* to 6.5 in *A. herba-alba.* The amount observed in *A. herba-alba* was significantly higher compared to the amount found in roots of other plant species (Table 3 and Table 4). For the shoot parts, the lowest accumulation of Ni up to 0.5 mg kg^−1^ DW is found in *R. rigosum* while the highest value up to 8.1 mg kg^−1^ DW was reached significantly in *A. herba-alba* followed by *C. bursa-pastoris* (4.4 mg kg^−1^ DW) and *S. hispanicus* (4.2 mg kg^−1^ DW) compared to other species (Table 3 and Table 4). According to Krämer [67], none of the plants sampled from both sites exceed phytotoxic Ni concentrations. This result from low concentration of Ni in soils. According to Reeves [77], none of plants species showed a Ni hyperaccumulation characteristic.

None of the plants harvested from Oued el Heimer and Touissite has the BCF > 1 (Table 5). However, eight plant species have translocation factors superior to 1 namely *C. libanotis* (1.27), *A. herba-alba* (1.24), *C. bursa-pastoris* (Oued el Heimer site) (1.24), *P. communis* (1.17), *C. althaeoides* (1.17), *S. hispanicus* L. (1.15), *C. bursa-pastoris* (Touissite site) (1.08), and *A. alopecuroides* (1.05) (Table 5). These plants have a good capacity to translocate Ni from roots to the shoots [14]. BAC values range from 0.05 in *H. incana* to 0.62 in *A. herba-alba*. According to Yoon et al. [16], none of these plant species has the potential to be used for phytostabilization (BCF > 1 and TF < 1) or phytoextraction (BCF, TF, and BAC > 1) of Ni.

#### 3.4.5. Lead (Pb)

Pb concentrations in plant roots ranged from 235.1 to 3785.7 mg kg^−1^ DW in *A. alopecuroides* and *S. tenacissima,* respectively (Table 3). This level of Pb in *S. tenacissima* was significantly higher (*p* < 0.05) to other plants species, followed by *P. communis*, and *A. herba-alba*. No considerable difference of Pb content was observed in the other plant species. Moreover, in eight plant species, the Pb amounts in roots were higher than those found in the shoots, indicating a low translocation rate from roots to shoots. Many studies reported Pb concentrations in different plant species growing from contaminated soils. Midhat et al. [8] and Brunetti et al. [78] recorded about 0.7 and 282.33 mg kg^−1^ DW of Pb in roots respectively of *Stipa austroitalica* and *Stipa capensis* Thunb. However, Nouri et al. [61] reported up to 1743 mg kg^−1^ DW of Pb in the root of *Reseda alba L* growing in Pb–Zn mine in Iran. These values are higher than those observed in the root of *R.a alba* (322.7 mg kg^−1^ DW) collected from Touissite site and lower than those found in *S. tenacissima* collected from Oued el Heimer site. The variation of Pb accumulation in the roots of *R. alba,* and the genus of *Stipa* can significantly be related to the physicochemical properties of soils.

In shoot, the concentrations of Pb vary from 47.1 mg kg^−1^ DW in *R. rigosum* to 4672. 2 mg kg^−1^ DW in *A. herba alba* (belonging to Asteraceae family) (Table 3). Pb content found in *A. herba alba* was significantly higher than those observed in *R. alba, C. libanotis, S. tenacissima,* and other plant species (Table 3 and Table 4). Many authors report high heavy metals hyperaccumulation ability in several plants belonging to the Asteraceae family. Nouri et al. reported high level of Pb up to 9017 mg kg-1 DW in shoots of *Scariola orientalis* growing in the vicinity of the Ahangaran lead–zinc mine (Hamedan, Iran) [61]. *Aster prorerus Hemsl,* a plant belonging to the Asteraceae family, collected near the smelter of the Baoshan mining project (China), showed a Pb concentration up to 3677 mg kg^−1^ DW in shoots [79]. In our study, four plant species namely *A. herba-alba* (4672.2 mg kg^−1^ DW), *R. alba* (1607.51 mg kg^−1^ DW), *C. libanotis* (1261.8 mg kg^−1^ DW), and *S. tenacissima* (1146.3 mg kg^−1^ DW) had a Pb concentration higher than 1000 mg kg^−1^ DW in their shoots, which exceeded the threshold for Pb hyperaccumulation in plants [14,75]. All studied plants sampled from both sites showed Pb concentrations higher than the phytotoxic one (0.6–28 mg kg^−1^) which means that they are tolerant to this metal.

The BCF value varied from 0.02 (*R. alba*) to 1.99 in *C. libanotis.* While the TF > 1 was recorded in six plant species namely *R. alba* (4.98), *A. herba alba* (2.67), *C. bursa-pastoris* (Oued el Heimer) (1.46), *A. alopecuroides* (1.24), *S. hispanicus* L. (1.21), and *C. libanotis* (1.03). The minimum and maximum values of BAC found in *R. rigosum* (0.001) and *C. libanotis* (2.06), respectively. According to Yoon et al. [16], three plant species from Oued el Heimer site have the potential for Pb phytoextraction. *C. libanotis* with BCF, TF, and BAC superior to 1, followed by *S. tenacissima,* which although it accumulates more than 1000 mg kg^−1^ of Pb in its shoots, does not meet the second criterion for hyperaccumulation (TF > 1). This plant remains a good candidate for Pb phytoextraction. The third plant is *A. herb-alba* which accumulates up to 1000 mg kg^−1^ in its shoots with a high translocation factor (2.67). *R. alba* from Touissite also can be used in Pb phytoextraction because it accumulates up to >1000 of Pb mg kg^−1^ in shoot parts with TF > 1. This result indicates that this plant species exhibited a good capacity to translocate Pb from roots to shoots. According to the criteria of phytostabilization (BCF > 1 and TF < 1), none of the collected plants has the potential for Pb phytostabilization [13].

#### 3.4.6. Zinc (Zn)

Zn content in shoots and roots were significantly different between species (Table 3 and Table 4). Zn concentrations in roots range between 135.6 and 637 mg kg^−1^ DW in *C. libanotis* and *S. tenacissima*, respectively (Table 3). However, no significant difference in Zn level in the roots of *S. tenacissima, A. herba-alba,* and *C. bursa-pastoris* (Touissite). The maximum Zn accumulated in shoot was 666.9 mg kg^−1^ DW in *C. bursa-pastoris* collected from Touissite. Zn content in *C. bursa-pastoris* shoots (from Touissite) was significantly higher compared to other 13 plant species. Nine plant species could accumulate concentrations of Zn higher than the phytotoxic levels (100–300 mg kg^−1^) in their tissues and were considered as Zn tolerant species, i.e., *R. alba*, *S. hispanicus*, *P. communis*, *C. bursa-pastoris* (from both site), *H. spinosissimum*, *R. rigosum*, and *S. tenacissima*, *A. herba-alba* (Table 3). Many authors recorded several plant species growing on contaminated soils that could accumulate a high concentration of Zn in different plant tissues, especially in roots. Nouri et al. reported 73 and 2938 mg Zn kg^−1^ DW in roots of *Reseda alba L* and *Reseda lutea L*, collected in the vicinity of a Pb–Zn mine in Iran [61]. Midhat et al. [8] reported 856.19 mg Zn kg^−1^ DW in *Stipa capensis Thunb* roots sampled from Sidi Bou-Othmane mining site (Marrakech city, Morocco). Regarding hyperaccumulation criteria, none of the plant species collected from the two areas exceeds the threshold of Zn hyperaccumulation > 10,000 mg kg^−1^ in their shoots and then cannot be considered as Zn hyperaccumulator [14].

The bioconcentration factor (BCF) varied from 0.01 in *H. incana* and *S. tenacissima* to 1.69 in *A. herba-alba.* Nine plant species have the TF > 1 namely *P. communis* (2.17), *R. alba* (1.83), *S. hispanicus* L. (1.46), *R. rigosum* (1.36), *C. libanotis* (1.19), *A. alopecuroides* (1.18), *C. bursa-pastoris* (Touissite) (1.13), *H. incana* (1.13), and *C. bursa-pastoris* (Oued el Heimer) (1.00) (Table 5). The highest BAC value was observed in *A. herba-alba* (1.04), and the lowest value was reported in S. *tenacissima* (0.001). According to Yoon et al. [16], none of the harvested plant species from both areas has the potential for phytoextraction of Zn (BCF, TF, and BAC > 1). *A. herba-alba,* with BCF > 1 and TF < 1, could be a suitable candidate for Zn phytostabilization to minimize Zn migration in soils [16,62].

#### 3.4.7. Antimony (Sb)

Sb content in shoots and roots were significantly different between species. The maximum Sb accumulated in roots was 167.7 mg kg^−1^ DW in *S. tenacissima* and the minimum was 1.6 mg kg^−1^ DW in *R. rigosum*. Sb content in roots of *S. tenacissima* was significantly higher than those observed in roots of other plants species (Table 3 and Table 4). *C. bursa-pastoris* (Touissite) and *A. herba-alba* accumulated the highest levels of Sb in their shoots (55.5 and 41.6 mg kg^−1^ DW, respectively), there was no significant between those two species, but a significant variation of Sb was observed in shoots between other plant species (Table 3). However, the lowest concentration of Sb 0.5 mg kg^−1^ DW in shoots was found in *R. rigosum* (Table 3). These plant species were grown in soils with maximum Sb concentrations of 247.1 and 242.8 mg kg^−1^ in Oued el Heimer and Touissite, respectively. Many authors indicate that the high levels of Sb in soils do not necessarily result in significant accumulation of Sb in plant tissues [80]. The concentration of this metal in plants depends on three principal factors, i.e., 1: the Sb bioavailability in soils; 2: Sb speciation; and 3: variations of coexisting ions in soils such as calcium (Ca) and phosphorus (P) [80]. In the present study, ten plant species accumulated more than phytotoxic concentration of Sb (5–10 mg kg^−1^) in their tissues and are expected as Sb tolerant plants, i.e., *R. alba, C. althaeoides, H. spinosissimum, P. communis, L. corniculatus, C. bursa-pastoris* (Touissite), *S. hispanicus* L., *S. tenacissima*, *A. herba-alba*, and *C. bursa-pastoris* (Oued el Heimer). All plant species did not accumulate >1000 mg kg^−1^ in their shoots, meaning that none of the harvested plants could be considered as Sb hyperaccumulator.

All plant species sampled from both sites exhibited a BCF less than 1 (Table 5). However, nine plant species showed a TF > 1 namely *R. alba* (3.55), *A. herba-alba* (3.2), *C. bursapastoris* (Touissite) (2.05), *S. hispanicus* L. (1.39), *A. alopecuroides* (1.19), *P. communis* (1.15), *H. incana* (1.2), and *C. libanotis* (1.13) and only one plant species, i.e., *A. herba-alba* (1.22) has recorded BAC > 1 (Table 5). According to Yoon et al. [16] none of the fourteen plant species is suitable for phytostabilization or phytoextraction of Sb.

In general, the majority of collected plant species from both prospected sites demonstrate a high ability to survive in this environment. However, all of them presented high heavy metal concentrations in different plant tissues that exceeded the phytotoxic levels without sustaining toxicity. BCF, TF, and BAC values indicate that only four plant species exhibited strong heavy metals tolerance and hyperaccumulation and then have a good potential to be used in phytoremediation strategies.

### 3.5. Plants Polymetallic Accumulation Ability

**Figure 4:**
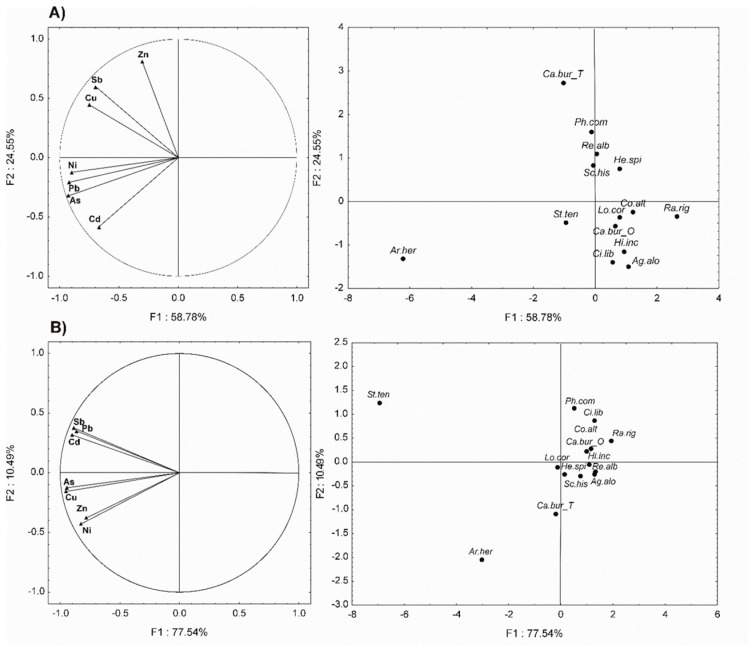
Plot of axes 1 and 2 of the principal components analysis (PCA) on the means of the metals concentrations separately in the shoots (**A**) and the roots (**B**) for the studied species. Abbreviation of species names: *Ag.Alo: Agathophora alopecuroides*; *Ci.lib: Cistus libanotus; Ca.bur_T:* *Capsella bursa-pastoris* Touissite site; *Ca.bur_O:* *Capsella bursa-pastoris* Oued El Heimer site; *Lo.cor: Lotus corniculatus; Ph.com: Phragmites communis; He.spi: Hedysarum spinosissimum; Co.alt: Convolvulus althaeoides; Re.alb: Reseda alba; Sc.his: Scolymus hispanicus; Ra.rig: Rapistrum rigosum; Hi.inc: Hirschfeldia incana; St.ten: Stipa tenacissima; Ar.her: Artemisia herba-alba.*

The biplot one-half of the principal components analysis (PCA) performed on metals concentrations in the shoots of the studied plant species (Figure 4A) explain 83.33% of the total variance. The F1 axis (58.78% of the total inertia) represent the species ability to accumulate different heavy metals simultaneously. It separated *Artemisia herba-alba* with high concentrations of As, Cd, Cu, Ni, and Pb in shoots from the other species (*Rapistrum rigosum, Convolvulus altheoides, Lotus corniculatus, Hirschfeldia incana,* etc.) with low concentrations of these metals in the shoots (Figure 4A). The F2 axis (24.55% of the total inertia) represents Zn and Sb concentration gradients (Figure 4A). It separates *Capsella bursa-pastoris* (from Touissite site), *Phragmites communis, Reseda alba, Scolymus hispanica, and* *Hedysarum spinosissimum* which are highly correlated with Zn and Sb concentrations in shoots, from the other species weakly correlated with Zn and Sb concentrations (Figure 4A). The clusters of plants species identified with PCA was confirmed by dendrogram obtained by ascending hierarchical classification (AHC) (Figure S1A).

The biplot one-half of the PCA performed on metals concentrations in roots of studied species (Figure 4B) explain 81.03% of the total variance. The F1 axis (77.54% for the total inertia) explains the major part of the information which represents the species ability to accumulate different heavy metals simultaneously (Figure 4B). According to this axis, *Stipa tenacissima* followed by *Artemisia herba-alba* presented a high correlation with As, Cd, Cu, Ni, Pb, Sb, and Zn indicating that these species have strong accumulation ability on roots of all studied metals (Figure 4B). The other group (*Capsella bursa-pastoris*“Touissite”, *Reseda alba*, and *Hedysarum* *spinosissimum*) form another cluster (Figure S1B). This shows that these species have a preferential accumulation capacity for specific metals (e.g., *Cistus libanotis* for Pb (1607.5 mg kg^−1^ DW); *Stipa tenacissima* for Pb (1146.3 mg kg^−1^ DW))

In our knowledge, the uptake and translocation of metal ions by the plants depends not only on the concentration of these elements in the soil, but also on metal plant affinity, plant species and metal speciation [81]. Indeed, biotic (microorganisms)and abiotic soil characteristics (physical and chemical properties), have a significant influence on heavy metals content in plants [82]. As well, there is often an antagonism between ions in plants. As an example, antagonistic decrease in the uptake and accumulation of heavy metals in plants, e.g., ions of Pb or Cd was observed with the increase of the concentration of trace elements in the soil, e.g., Zn [83]. The potential antagonism between Pb and Zn occurs only in the case of some plants. According to our results, native wild plants *Stipa tenacissima* and *Artemisia herba-alba* showed cooperative accumulation of all metals in roots. Interestingly, *Artemisia herba-alba* demonstrated strong translocation capacity in shoots for As, Cd, Cu, Ni, and Pb.

### 3.6. Suitable Plant Species for Use in Phytoremediation Strategies

#### 3.6.1. *Stipa tenacissima* Suitable Plant for Cd/Cu Phytostabilization and Pb Phytoextraction

Among all plant species, only *Stipa tenacissima* from Oued el Heimer presented a high amount of Cd and Cu up to 241.2 mg kg^−1^ DW and 237.9 mg kg^−1^ DW, in roots respectively, with TF < 1. *Stipa tenacissima* is a perennial plant belonging to Poaceae family, commonly known as “Halfa” [84]. This species has already identified in other mining sites. Some authors reported that *Stipa tenacissima* species from Zaida mining site (Morocco) could accumulate up to 58.4 and 41.8 mg kg^−1^ DW of Cu in their roots and shoots, respectively [85]. Other species of genus *Stipa* from Sidi Bou-Othmane mining site (Marrakech, Morocco) accumulate 50.88 mg kg^−1^ DW of Cu and 0.87 mg kg^−1^ DW of Cd in their roots [8], with roots without being affected. Regarding the BCF and TF values, *Stipa tenacissima* presents a hyper tolerance characteristic with an excellent capacity to accumulate Cd and Cu in their roots. This native plant could be a suitable candidate for Cd/Cu phytostabilization.

*Stipa tenacissima* also accumulates up to 1226.9 mg kg^−1^ of Pb in their shoots. This concentration is higher than 193.2 mg kg^−1^ of Pb recorded in *Stipa tenacissima* plants harvested at Zaida Pb mining site (high Moulouya, Morocco) [86]. Our result showed that Pb accumulated in *Stipa tenacissima* shoots exceeds the hyperaccumulation thresholds, more than 1000 mg Pb/kg DW. Since the roots accumulated more Pb up to 3785.7 mg kg^−1^ than the shoots (TF < 1), the term hyperaccumulator is not fully appropriate. However, it appears that *Stipa tenacissima* exhibits a good Pb accumulation capacity and can be a suitable candidate for Pb phytoextraction.

#### 3.6.2. *A. herba-alba* Suitable Plant for Cu/Zn Phytostabilization and Pb Phytoextraction

The genus *Artemisia* belongs to Asteraceae family, that include many species known as accumulators of several metals [86,87,88]. In the present study, high amounts of Cu up to 203.1 mg kg^−1^ DW and Zn up to 576.9 mg kg^−1^ DW have been observed in the roots of *A. herba-alba*. The Cu and Zn concentration accumulated by *A. herba-alba* roots were higher than those reported by Baghdad et al. [85] at Zaida mining site (56.2 mg kg-^1^ DW of Cu and 133.9 mg kg-^1^ DW of Zn). Some *Artemisia* species are widespread on contaminated sites and have a large biomass production and high ability to accumulate heavy metals, especially Zn [88]. Alirzayeva et al. [88] reported up to 200.71 mg kg-^1^ DW of Zn in roots of *A. scoparia,* and up to 112.24 mg kg-^1^ DW in roots of *A. fragrans* was reported by Ashraf et al., [89]. In our study, *A. herba-alba* characterized by BCF > 1 and a high amount of Zn and Cu in its roots and is then a good candidate for Cu/Zn phytostabilization.

*Artemisia herba-alba* accumulates also a large amount of Pb in roots (1748.7 mg kg^−1^ DW) and shoots (4672.2 mg kg^−1^ DW). These amounts are higher than those recorded in the same plant species growing at different Pb mining sites in Morocco (704.0 and 660.0 mg kg^−1^ DW of Pb in its roots and shoots, respectively) [85]. *Artemisia lancangensis,* another plant from *Artemisia genus* growing at the lead–zinc mine area in China, accumulates 12.83 mg kg^−1^ DW of Pb in their shoots [90]. Other authors recorded 892 mg kg^−1^ DW in root and 834 mg kg^−1^ DW Pb in the shoot of *Artemisia lactiflora* Wall from Beiya Pb mine area, China [91], 11.58 mg kg^−1^ DW in the shoot of *Artemisia vulgaris* growing near mining and industry in upper Silesia (southern Poland) [92] and 375, 446, 539 and 3677 mg kg^−1^ DW of Pb in *Artemisia taurica*, *Artemisia argyi, Artemisis japonica,* and *Aster prorerus,* respectively growing on polluted soils, with Pb concentrations ranging from 568 to 49,294 mg kg^−1^ at the Baoshan mining area (China) [79]. The higher amount of Pb in the shoot of *Artemisia herba-alba* can be due to the strong capacity of metal uptake and an enhanced xylem loading capacity for metals [79]. According to previous studies, this is the first time that *A. herba-alba* is identified as hyper-tolerant and hyperaccumulator of Pb. This is not a general characteristic of Asteraceae family. Our result showed that Pb accumulated in *Artemisia herba-alba* exceeds the hyperaccumulation threshold of 1000 mg Pb kg^−1^ DW. Since the shoots accumulated more Pb than roots (TF = 2.67), the term hyperaccumulator is fully appropriate, which means that *A. herba-alba* evolved high Pb transport ability from the roots to the shoots, and can be suggested as a good candidate for Pb phytoextraction.

#### 3.6.3. *Reseda alba* a Powerful Plant Species for Pb Phytoextraction

*R. alba* belonging to the Resedaceae family, was known for its potential to accumulate Pb, Zn, and Cd in roots and shoots [13,93]. In our study, this species accumulates 1607.5 mg kg^−1^ DW of Pb in their shoots, with high TF = 4.98. However, Nouri et al. [61] indicate that *Reseda alba* from the Ahangaran lead–zinc mine in Hamedan, Iran (soil: 9535 mg kg^−1^ of Pb), can accumulate up to 703 mg kg^−1^ DW of Pb in aboveground parts. The change in soil conditions can have many impacts on the speciation and distribution of metals [41]. For example, pH plays an important role in the solubility and mobility of heavy metals. Bliefert et al. [39] reported that pH more than 7.5 decreases significantly the mobility of Pb while the mobility of this metal is greater in acidic soils. To our knowledge, this is the first time that *Reseda alba* is described as high Pb accumulator since its Pb content exceeded the hyperaccumulator threshold of 1000 mg kg^−1^ DW and TF > 1. *R. alba* then could be an excellent candidate for the Pb phytoextraction.

#### 3.6.4. *Cistus libanotis* a Good Candidate for Pb Phytoextraction

*Cistus libanotis* is a fast-growing perennial plant with high biomass [94]. In the present study, *Cistus libanotis* can accumulate up to 1261.8 mg kg^−1^ DW of Pb in shoots, with TF > 1. These results confirmed that *Cistus libanotis* is a good candidate for use in phytoremediation, especially in phytoextraction. *Cistus libanotis* was reported previously by our team as an excellent plant for Pb phytoextraction [95]. Plants belonging to *Cistus* genus show high plasticity. They can grow both in contaminated and non-contaminated soils being tolerant to high concentrations of trace elements, like As and Pb [96,97]. *C. albidus* and *C. salviifolius* respectively accumulate higher amounts of Pb up to 4831 and 2896 mg kg^−1^ of Pb in their roots than in their shoots and were considered suitable for phytostabilization of Pb contaminated soils [94]. The same study indicates that *Cistus libanotis* can accumulate a large amount of Pb in its roots and shoots (10,340 and 2055 mg Pb kg^−1^ DW, respectively) when grown in hydroponic conditions [94]. Santos et al. [98] suggest that *C. ladanifer* is suitable for phytostabilization of mine soils rich in Pb, As, Cu, and Zn. However, metal accumulation is not a general pattern of this genus. In the present study, *Cistus libanotis* collected from Oued el Heimer site can be a good candidate for Pb hyperaccumulation.

## 4. Conclusions

Long term mining activities in Oued el Heimer and Touissite areas in eastern Morocco has caused strong heavy metal contaminations related to massive hazardous and poorly managed waste deposits. Soils are characterized by high levels of contamination especially Pb, Cd, Zn, and the critical metal Sb. Native plant species identified in these areas present a high ability to tolerate and accumulate several metals in their tissues. Among the 14 plant species collected from these areas, only four plants are identified as Pb hyperaccumulators, namely *Reseda alba, Stipa tenacissima, Artemisia herba-alba,* and *Cistus libanotis.* Regarding the high amounts of metals in their roots and shoots and BCF, TF, and BAC values, these plant species, classified as hyper-tolerant with a strong capacity to accumulate several metals, are promising native plants for Pb phytoextraction. *Stipa tenacissima* and *Artemisia herba-alba* have the potential to be used in phytostabilization for Cd/Cu and Cu/Zn, respectively. Further investigations will be led to increase the value of metal-phytomining potential from Oued el Heimer and Touissite soils using suitable selected plants species.

## Figures and Tables

**Table 1:** Soil proprieties (pH, EC: Electrical conductivity, OM: Organic Matter) and range concentrations (mg kg^−1^ DW) of As, Cd, Cu, Ni, Pb, Sb, and Zn in soils sampled from studied areas.

Site		As	Cd	Cu	Ni	Pb	Zn	Sb	PI	pH	EC (mS/cm)	% OM
Oued el Heimer	Concentration range in soils (mg kg^−1^)	18.7–466	32–280	35–592	12–44.4	611–12,461	318–43,540	33.9–247.1	3.62–67.9	6.4–7.51	0.40–2.28	0.41–80%
	Enrichment factor (EF)	15–364.8	233–3656	5.3–98.8	2.5–8.2	131.7–2995	26.5–2749	285.7–2393				
Touissite	Concentration range in soils (mg kg^−1^)	43–82.9	15–36	328–1405	9–16	6445–18,324	2096–5387	96.7–242.8	14.0–38.5	6.9–7.7	0.23–0.38	0.28–2.07%
	Enrichment factor (EF)	22.2–52.5	133–410.4	29.5–166.5	1.7–2.3	833.5–2758	115.2–1092	550.3–1877				

**Table 2:** List of sampled plants in the vicinity of the studied sites.

Site	Plants	Family	Life Span
Touissite	*Reseda alba*	Resedaceae	Annual
	*Convolvulus althaeoides*	Convolvulaceae	Perennial
	*Hedysarum spinosissimum*	Fabaceae	Annual
	*Phragmites communis*	Poaceae	Perennial
	*Lotus corniculatus*	Fabaceae	Perennial
	*Capsella bursa-pastoris*	Brassicaceae	Annual
	*Scolymus hispanicus*	Asteraceae	Perennial
	*Rapistrum rigosum*	Brassicaceae	Annual
Oued el Heimer	*Cistus libanotis*	Cistaceae	Perennial
	*Agathophora alopecuroides*	Amaranthaceae	Perennial
	*Hirschfeldia incana*	Brassicaceae	Perennial
	*Stipa tenacissima*	Poaceae	Perennial
	*Artemisia herba-alba*	Asteraceae	Perennial
	*Capsella bursa-pastoris*	Brassicaceae	Annual

**Table 3:** Pair-wise differences of heavy metals concentrations (mg kg^−1^ DW) in the roots/shoots of plants collected from the studied sites in the oriental region of Morocco.

		As		Cd		Cu		Ni		Pb		Zn		Sb	
Site	Plants	Root	Shoot	Root	Shoot	Root	Shoot	Root	Shoot	Root	Shoot	Root	Shoot	Root	Shoot
Touissite	*R. alba*	(2.6) ^a^	(6.3) ^bc^	(2.1) ^a^	(4.1) ^ab^	(45.1) ^ab^	(46.1) ^de^	(2.9) ^abc^	(2.0) ^ab^	(322.7) ^a^	(1607.5) ^f^	(254.3) ^a^	(465.5) ^c^	(6.1) ^ab^	(21.7) ^abcd^
	*C. althaeoides*	(2.4) ^a^	(5.1) ^abc^	(2.9) ^a^	(1.3) ^a^	(59.7) ^ab^	(35.4) ^bcd^	(1.89) ^ab^	(2.2) ^ab^	(714.3) ^abc^	(426.3) ^abcd^	(286.1) ^a^	(207.4) ^ab^	(16.0) ^bcd^	(13.6) ^abc^
	*H. spinosissimum*	(6.0) ^a^	(3.6) ^ab^	(8.7) ^a^	(3.9) ^ab^	(58.5) ^ab^	(32.6) ^bcd^	(4.1) ^abc^	(2.3) ^ab^	(983.6) ^abc^	(253.9) ^ab^	(347.0) ^ab^	(314.0) ^abc^	(35.1) ^e^	(33.1) ^cde^
	*P. communis*	(3.8) ^a^	(5.0) ^abc^	(2.7) ^a^	(2.0) ^ab^	(66.7) ^ab^	(65.8) ^ef^	(2.2) ^ab^	(2.6) ^ab^	(2305.7) ^d^	(720.4) ^bcde^	(198.9) ^a^	(432.5) ^c^	(22.1) ^cd^	(25.5) ^abcde^
	*L. corniculatus*	(5.3) ^a^	(3.0) ^ab^	(3.0) ^a^	(1.8) ^a^	(117.3) ^bc^	(39.6) ^bcd^	(4.1) ^abc^	(3.3) ^ab^	(1493.0) ^bcd^	(832.4) ^bcde^	(276.6) ^a^	(170.5) ^a^	(27.0) ^de^	(14.8) ^abcd^
	*C. bursa-pastoris*	(3.8) ^a^	(5.9) ^bc^	(4.4) ^a^	(4.2) ^ab^	(65.7) ^ab^	(49.0) ^def^	(4.1) ^abc^	(4.4) ^b^	(849.6) ^abc^	(733.8) ^bcde^	(587.0) ^b^	(666.9) ^d^	(27.0) ^de^	(55.5) ^e^
	*S. hispanicus* L.	(3.6) ^a^	(4.7) ^abc^	(5.2) ^a^	(5.4) ^ab^	(48.8) ^ab^	(44.3) ^cde^	(3.6) ^abc^	(4.2) ^b^	(798.7) ^abc^	(972.7) ^de^	(307.9) ^a^	(452.0) ^c^	(12.3) ^bc^	(17.2) ^abcd^
	*R. rigosum*	(1.3) ^a^	(0.3) ^a^	(1.7) ^a^	(1.8) ^a^	(13.6) ^a^	(10.0) ^a^	(1.3) ^a^	(0.5) ^a^	(454.8) ^ab^	(47.1) ^a^	(239.9) ^a^	(326.8) ^abc^	(1.6) ^a^	(0.5) ^a^
Oued el Heimer	*C. libanotis*	(3.4) ^a^	(9.7) ^c^	(22.7) ^a^	(16.2) ^bc^	(23.6) ^ab^	(25.5) ^abcd^	(2.4) ^ab^	(3.1) ^ab^	(1219.2) ^abcd^	(1261.8) ^ef^	(135.6) ^a^	(161.7) ^a^	(8.3) ^ab^	(9.4) ^abc^
	*A. alopecuroides*	(9.1) ^a^	(4.6) ^abc^	(4.9) ^a^	(18.0) ^bcd^	(26.0) ^ab^	(16.3) ^ab^	(4.0) ^abc^	(4.2) ^abc^	(235.1) ^a^	(293.8) ^ab^	(142.5) ^a^	(168.2) ^a^	(5.2) ^ab^	(6.2) ^abc^
	*H. incana*	(6.0) ^a^	(6.6) ^bc^	(25.8) ^a^	(32.9) ^d^	(37.3) ^ab^	(19.5) ^ab^	(2.9) ^abc^	(2.3) ^ab^	(441.3) ^ab^	(343.7) ^abc^	(273.4) ^a^	(309.7) ^abc^	(5.3) ^ab^	(6.4) ^ab^
	*S. tenacissima*	(59.3) ^b^	(19.6) ^d^	(241.2) ^b^	(28.9) ^cd^	(237.9) ^dc^	(29.7) ^abcd^	(6.4) ^bc^	(3.8) ^ab^	(3785.7) ^e^	(1146.3) ^ef^	(637.0) ^b^	(322.9) ^abc^	(167.7) ^f^	(32.1) ^bcde^
	*A. herba-alba*	(48.7) ^b^	(50.7) ^e^	(50.2) ^a^	(56.2) ^e^	(203.1) ^cd^	(72.3) ^f^	(6.5) ^c^	(8.1) ^c^	(1748.7) ^cd^	(4672.2) ^g^	(577.0) ^b^	(357.0) ^bc^	(13.0) ^bc^	(41.6) ^de^
	*C. bursa-pastoris*	(4.5) ^a^	(5.1) ^abc^	(41.4) ^a^	(30.0) ^cd^	(28.7) ^ab^	(18.2) ^abc^	(1.7) ^ab^	(2.1) ^ab^	(629.7) ^abc^	(920.9) ^cde^	(395.3) ^ab^	(396.1) ^bc^	(8.7) ^ab^	(12.9) ^abcd^
Phytotoxic concentrations of metals [66,67]		2–80		0.1–3		20–30		10–50		0.6–28		100–300		5–10	
Hyperaccumulation threshold [67]		>1000		>100		>1000		>1000		>1000		>10,000		>1000	

**Explanation:** The values in the table represent the average of heavy metals contents in roots & shoots for each plant species. The averages followed by the same letters do not differ significantly.

**Table 4:** Results of variance analysis (One-way ANOVA) comparing the difference in metal concentration among species (dF = 13).

	Shoots		Roots	
	*F*	*p*	*F*	*p*
As	111.03	<0.0001	46.19	<0.0001
Cd	29.74	<0.0001	19.95	<0.0001
Cu	14.82	<0.0001	10.53	<0.0001
Ni	6.91	<0.0001	3.06	0.0180
Pb	101.65	<0.0001	14.69	<0.0001
Zn	18.43	<0.0001	8.16	<0.0001
Sb	7.54	<0.0001	250.93	<0.0001

**Table 5:** Bioconcentration factor (BCF), Translocation factor (TF) and Biological accumulation coefficient (BAC) for the studied plants species.

		Trace Metal Elements																				
		As			Cd			Cu			Ni			Pb			Zn			Sb		
**Site**	Plants	BCF	TF	BAC	BCF	TF	BAC	BCF	TF	BAC	BCF	TF	BAC	BCF	TF	BAC	BCF	TF	BAC	BCF	TF	BAC
Touissite	*R. alba*	0.04	**2.37**	0.10	0.08	**1.91**	0.15	0.11	**1.02**	0.11	0.32	0.66	0.21	0.02	**4.98**	0.12	0.04	**1.83**	0.08	0.04	**3.55**	0.15
	*C. althaeoides*	0.03	**2.12**	0.07	0.08	0.62	0.04	0.12	0.59	0.07	0.13	**1.17**	0.15	0.09	0.59	0.05	0.05	0.72	0.03	0.10	0.85	0.08
	*H. spinosissimum*	0.13	0.59	0.08	0.57	0.44	0.24	0.17	0.55	0.09	0.25	0.57	0.14	0.15	0.25	0.03	0.10	0.90	0.10	0.36	0.94	0.34
	*P. communis*	0.06	**1.31**	0.08	0.07	0.73	0.05	0.04	0.99	0.04	0.18	**1.17**	0.20	0.12	0.31	0.03	0.04	**2.17**	0.08	0.09	**1.15**	0.10
	*L. corniculatus*	0.08	0.57	0.04	0.19	0.55	0.1	0.14	0.33	0.05	0.25	0.80	0.2	0.08	0.55	0.05	0.13	0.60	0.08	0.12	0.54	0.06
	*C. bursa-pastoris*	0.04	**1.53**	0.07	0.12	0.95	0.11	0.09	0.74	0.06	0.27	**1.08**	0.29	0.09	0.88	0.08	0.13	**1.13**	0.15	0.11	**2.05**	0.22
	*S. hispanicus* L.	0.06	**1.32**	0.07	0.20	**1.03**	0.21	0.14	0.90	0.12	0.27	**1.15**	0.32	0.10	**1.21**	0.12	0.1	**1.46**	0.14	0.07	**1.39**	0.1
	*R. rigosum*	-	0.2	-	-	0.8	-	-	0.7	-	-	0.4	-	-	0.1	-	-	**1.36**	-	-	0.3	-
Oued el Heimer	*C. libanotis*	0.18	**2.87**	0.51	0.60	0.71	0.42	0.67	**1.08**	0.71	0.17	**1.27**	0.22	**1.99**	**1.03**	**2.06**	0.42	**1.19**	0.50	0.24	**1.13**	0.27
	*A. alopecuroides*	0.48	0.50	0.24	0.15	**3.67**	0.56	0.74	0.62	0.05	0.23	**1.05**	0.24	0.38	**1.24**	0.48	0.07	**1.18**	0.08	0.03	**1.19**	0.04
	*H. incana*	0.01	**1.10**	0.01	0.09	**1.27**	0.11	0.06	0.52	0.03	0.06	0.79	0.05	0.03	0.77	0.03	0.01	**1.13**	0.01	0.02	**1.2**	0.03
	*S. tenacissima*	0.14	0.32	0.04	**2.72**	0.11	0.32	**1.55**	0.12	0.19	0.40	0.59	0.23	0.51	0.30	0.09	0.01	0.50	0.00	0.89	0.19	0.17
	*A. herba-alba*	0.86	**1.04**	0.90	0.80	**1.11**	**1.10**	**3.63**	0.35	**1.29**	0.54	**1.24**	0.62	0.48	**2.67**	**1.30**	**1.69**	0.61	1.04	0.38	**3.2**	**1.22**
	*C. bursa-pastoris*	0.01	**1.13**	0.01	0.86	0.72	0.16	0.05	0.63	0.03	0.07	**1.24**	0.09	0.05	**1.46**	0.07	0.03	**1.00**	0.03	0.03	**1.48**	0.05

**Bold values** indicate BCF, TF and BAC values greater than 1.0.

## Supplementary Materials

The following are available online at https://www.mdpi.com/2223-7747/9/11/1458/s1, Table S1: Heavy metal concentrations and reference element (mgKg^−1^ DW) in the rhizospheric soils samples collected from studied area in the oriental region of Morocco., Figure S1. Dendrogram obtained by Ascending Hierarchical Classification (AHC) carried out on the metals concentrations in the shoots (A) and the roots (B) of the studied plants.
