# Supplementary material for: Screening of Native Plants Growing on a Pb/Zn Mining Area in Eastern Morocco: Perspectives for Phytoremediation
Source: Plants (Basel). 2020 Oct 29;9(11):1458. doi: 10.3390/plants9111458 (PMC7693513; doi:10.3390/plants9111458)
Supplement: Supplementary file 1 [file plants-09-01458-s001.pdf]

Table S1: Heavy metal concentrations and reference element (mg kg^{-1} DW) in the rhizospheric soils samples collected from studied sites in the oriental region of Morocco.

		Heavy metals							
**Site**	**Plants**	As	Cd	Cu	Ni	Pb	Zn	Sb	Sc (ref. element)
**Touissite**	*R. alba*	61 ±11.7	26.2 ±2.3	402 ±118	9 ±2.6	13487.6 ±2364.6	5387.1 ±886.9	139.3 ±27.0	2.0
	C. althaeoides	71.6 ±2.2	26.3 ±3.2	479.7 ±49	14.3 ±1.7	8218.3 ±1807.2	5366.5 ±63.1	158.7 ±41.8	2.6
	H. spinosissimum	43 ±13.5	15 ±2.3	328 ±22.3	15.7 ±2.0	6445.1 ±707.6	3264.1 ±900.5	96.7 ±10.5	3.4
	P. communis	59.7 ±11.9	36.5 ±10.6	1405.8 ±409.5	12.3 ±2.1	18324.5 ±1051.4	2676.8 ±107.9	242.8 ±91.7	2.5
	L. corniculatus	62.7 ±9.4	16.9 ±1.1	786.4 ±194.1	16 ±1.6	12223.8 ±158.6	2096.4 ±646.9	224.2 ±68.5	3.0
	C. bursa-pastoris	82.9 ±24.4	35.8 ±3.0	706.7 ±155.7	14.7 ±1.6	8571.5 ±1127.1	4358.3 ±226.2	236.9 ±39.1	2.6
	S. hispanicus L	59.4 ±9.6	25.1 ±3.5	351.5 ±35.6	13 ±1.5	7792.9 ±2656.8	3049.1 ±324.3	168.1 ±32.2	3.1
	R. rigosum	nd	nd	nd	nd	nd	nd	nd	nd
**Oued el Heimer**	*C. libanotis*	18.7 ±8.8	37.8 ±10.4	35 ±18.2	14 ±2.9	611 ±99.6	318 ±82.8	51.4 ±21.2	2.0
	A. alopecuroides	133.4 ±45.5	32 ±12.9	314.8 ±91.5	17.3 ±2.1	6146.2 ±1675.3	2018.3 ±1176.5	152.7 ±44.0	2.4
	H. incana	466 ±127	280 ±62.5	589.4 ±130.8	44.4 ±3.1	12240.4 ±925.3	18232.3 ±2400.2	212.2 ±54.1	2.2
	S. tenacissima	424.5 ±34.1	88.6 ±8.4	152.5 ±59	16.1 ±0.7	7284 ±1468.8	43540 ±196.6	188.0 ±81.0	2.6
	A. herba-alba	56.3 ±8.3	50.8 ±10.1	55.8 ±14.9	12 ±1.3	3588.6 ±826.9	340.4 ±11.5	33.9 ±1.8	1.9
	C. bursa-pastoris	261.2 ±62.7	47.9 ±21.3	592 ±115	22.8 ±0.7	12461.3 ±1165.7	13068.3 ±209	247.1 ±6.0	1.8
	**Average crust**	6.83	0.41	38.9	29	27	29	0.67	11.7

Nd: not detected
The values in the table represent "mean ± standard deviation"

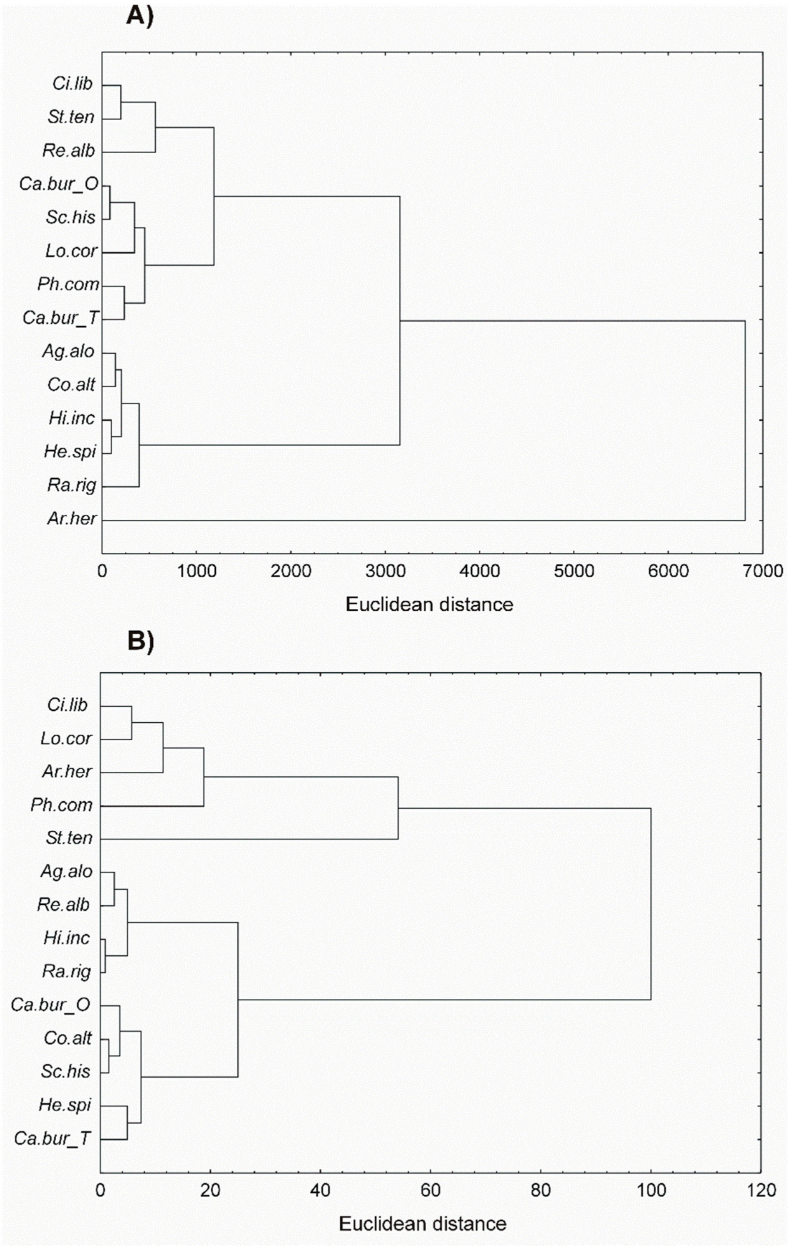

Figure S1. Dendrogram obtained by Ascending Hierarchical Classification (AHC) carried out on the metals concentrations in the shoots (A) and the roots (B) of the studied plants. Abbreviation of species names: *Ag.Alo : Agathophora alopecuroides*; *Ci.lib : Cistus libanotus; Ca.bur_T: Capsella bursa-pastoris* Touissite site*; Ca.bur_O: Capsella bursa-pastoris* Oued El Heimer site; *Lo.cor: Lotus corniculatus; Ph.com: Phragmites communis; He.spi: Hedysarum spinosissimum; Co.alt: Convolvulus althaeoides; Re.alb: Reseda alba; Sc.his: Scolymus hispanicus; Ra.rig: Rapistrum rigosum; Hi.inc: Hirschfeldia incana; St.ten: Stipa tenacissima; Ar.her: Artemisia herba-alba.*
